# The Effectiveness of a UAV-Based LiDAR Survey to Develop Digital Terrain Models and Topographic Texture Analyses

**DOI:** 10.3390/s23146415

**Published:** 2023-07-14

**Authors:** Piotr Bartmiński, Marcin Siłuch, Waldemar Kociuba

**Affiliations:** Institute of Earth and Environmental Sciences, Maria Curie-Skłodowska University in Lublin, 20-031 Lublin, Poland; piotr.bartminski@mail.umcs.pl (P.B.); marcin.siluch@mail.umcs.pl (M.S.)

**Keywords:** LiDAR-UAV survey, laser sensors efficiency comparison, DTM output from LiDAR-UAV data

## Abstract

This study presents a comparison of data acquired from three LiDAR sensors from different manufacturers, i.e., Yellow Scan Mapper (YSM), AlphaAir 450 Airborne LiDAR System CHC Navigation (CHC) and DJI Zenmuse L1 (L1). The same area was surveyed with laser sensors mounted on the DIJ Matrice 300 RTK UAV platform. In order to compare the data, a diverse test area located in the north-western part of the Lublin Province in eastern Poland was selected. The test area was a gully system with high vegetation cover. In order to compare the UAV information, LiDAR reference data were used, which were collected within the ISOK project (acquired for the whole area of Poland). In order to examine the differentiation of the acquired data, both classified point clouds and DTM products calculated on the basis of point clouds acquired from individual sensors were compared. The analyses showed that the largest average height differences between terrain models calculated from point clouds were recorded between the CHC sensor and the base data, exceeding 2.5 m. The smallest differences were recorded between the L1 sensor and ISOK data—RMSE was 0.31 m. The use of UAVs to acquire very high resolution data can only be used locally and must be subject to very stringent landing site preparation procedures, as well as data processing in DTM and its derivatives.

## 1. Introduction

The development of advanced measurement technologies in recent decades has created new perspectives in terms of spatial data acquisition capabilities. This is particularly true for acquisition techniques based on remote sensing solutions related to the retrieval of reliable information about physical objects and their surroundings, by means of registration, measurement and interpretation of images or their digital representations obtained through sensors that are not in direct contact with these objects [1,2].

Remote sensing may be divided according to different criteria [3]. Based on the distance from the studied object, remote sensing is divided into: (i) satellite-based; (ii) medium-altitude (airborne and unmanned aerial vehicle—UAV) and (iii) ground-based. Remote sensing data recording systems are either passive (when they record solar radiation reflected from objects or emitted by an object), or active (when they emit their own electromagnetic radiation which, after contact with an object, is recorded by a sensor). Remote sensing systems are also characterised by different resolutions: (a) spatial, defining the size of the smallest object distinguishable in the image recorded by the sensor, (b) spectral, referring to the specific wavelength range of electromagnetic radiation recorded by the sensor, (c) radiometric, which specifies the number of distinguishable radiation levels, and (d) temporal, defining the frequency of information acquisition.

The studies aimed at acquiring high-precision field data use sensors that emit electromagnetic radiation of different wavelengths. Of all the light measurement techniques (light detection and ranging, LiDAR), sensors using laser radiation are most commonly used to survey the physical characteristics of environmental features. The most commonly used types of survey in this group of techniques are airborne laser scanning (ALS) and terrestrial laser scanning (TLS). These are 3D imaging technologies that use laser beams to scan and measure physical objects of the environment [4]. By emitting pulses of a laser beam and then measuring the parameters of the reflected beam, ALS and TLS systems can determine the distance and angles between points in a given space. In technological terms, the time of flight of the laser pulse is measured. On the basis of the time measurement, it is possible to calculate the range and thus the distance between the emitter (scanner) and the measured object that generates the backscatter echo. Information about the geometry of the active surface can thus be obtained [5,6]. This data can be used to create a 3D model of the environment and provide detailed information about a given area. Laser sensor-based scanners are widely used in fields such as engineering and architecture, archaeology, forestry and terrain topography mapping [7]. TLS enables high-precision and high-resolution data acquisition, but for technological reasons it is only qualified for spatially limited 1 km^2^, local applications. Traditional ALS (based on aircraft and helicopter platforms), is much more versatile and has many times greater spatial coverage [8,9], but incurs high data acquisition costs.

Medium- and low-altitude remote sensing solutions based on active LiDAR sensors using a BSP as a carrier platform have become increasingly common in recent years [10]. Using this type of system combines the advantages and reduces the disadvantages of other solutions.

LiDAR-UAV data are characterised by high or very high spatial resolution. Accurate ground surface data can also be acquired in terrain with limited accessibility and/or dense vegetation cover [11]. Compared to satellite data, they offer much higher resolution and their acquisition is also independent of cloud conditions [12]. Brede et al. [13] showed that ALS produces data of comparable quality to TLS. Alternatively, high-resolution aerial RGB imagery may be acquired, which is much cheaper and easier to process into a point cloud than using lidar sensors [14]. Photogrammetric techniques can be a valuable alternative for vegetation surveys [15], but for ground surface analyses their use is limited by the type and density of vegetation cover [16]. Lidar sensors can be placed on aircraft, but a significant barrier to their widespread use is the high cost of equipment and platform maintenance, as well as the need to plan data acquisition in advance, taking into account weather conditions [17]. Comparable advantages to ALS are offered by the more cost-effective, accessible and easier to plan LiDAR-UAV. A certain limitation for LiDAR-UAV systems is still the relatively high cost of the system [18], and alternatives are proposed [18,19]).

LiDAR-UAVs are used in many applications, including 3D mapping [20] forestry [21], agriculture [22] and infrastructure monitoring [23]. They are also used where TLS is used [24]; nevertheless, LiDAR-UAV can be used to scan a larger area in much less time than with ground-based methods [25].High-resolution digital terrain models (DTMs) can be created from LiDAR-UAV survey data. These can form the basis of various types of geomorphometric analyses [26], landforms measurements and land surface change analyses [27]. LiDAR-derived DTMs are currently used in many scientific fields [28,29] and industrial–technical activities [30,31,32]. They provide information that is crucial or even necessary for environmental management in the broadest sense [33]. They can be used for modelling complex hydrological phenomena, related, for example, to the determination of the surface runoff of rainwater, the definition of catchment boundaries and drainage networks, the determination of soil moisture, and hydraulic modelling for flood risk assessment [34,35,36,37]. Other examples include modelling geohazards, including avalanche hazards, or forest fire hazards in mountainous areas [38,39]. Geomorphological applications include qualitative and quantitative descriptions of landforms [9,40,41,42]. They are also increasingly used in geological studies [43,44]. Land surface modelling is particularly relevant for assessing water erosion risk [45]. Of the other applications, it is worth mentioning the modelling of the spread of industrial pollutants or contamination that may be associated with transport events, for example, as well as the potential for use in forestry and agriculture [11,46].

In recent years, the market offer of LiDAR-UAV solutions has expanded significantly. Based on data received from various manufacturers, LiDAR sensors dedicated to UAVs offer very high accuracy and repeatability of measurements. On the other hand, Pilarska et al. [47] points out that particular LiDAR-UAV solutions can generate systematic error, mainly due to the imperfection of the inertial unit, which records the system’s operating angles (roll, pitch, yaw); however, the errors are estimable. For technological reasons, it has to be assumed that measurements made with the various sensors may further differ from each other. In the last few years, studies have been undertaken to directly compare specific laser scanners with each other [48]. Data acquired with LiDAR-UAV techniques have also been validated by reference to products acquired with other techniques [12,49]. There are still few studies available that actually illustrate product differences between specific platforms. The present work fills a gap in the mentioned area.

The aim of this study is to directly compare altitude data from different LiDAR-UAV systems and data acquired using aircraft, to demonstrate whether the test results obtained are sufficiently accurate to achieve the stated goal, and whether the platform provides the ability to reliably measure a given environmental element. Derived products developed from three popular LiDAR-UAV systems were used in relation to airborne laser scanning data to better recognise the variation between the different systems. The authors assumed the role of the end user, relying on off-the-shelf system solutions currently offered on the market.

## 2. Materials and Methods

**Figure 1:**
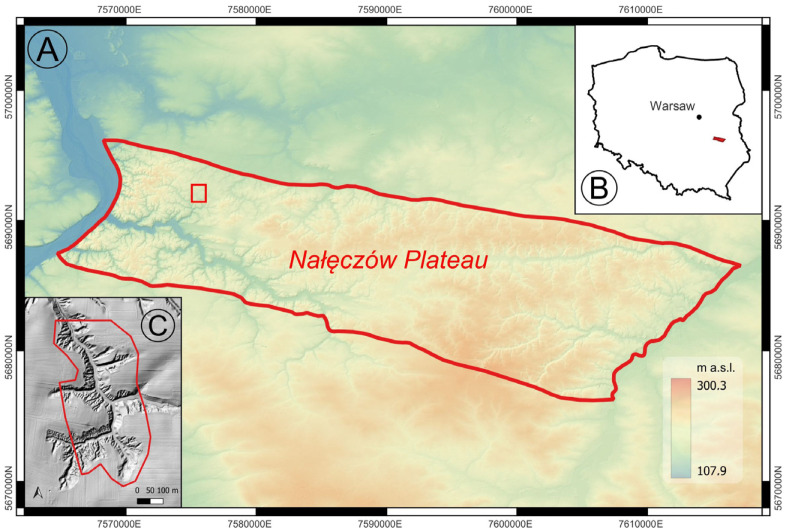
Location of the study area: (**A**) Nałeczów Plateau subregion with border of area of interest (AoI, red rectangle); location of investigated area in Poland (**B**); Area of interest (**C**). Boundaries of physical-geographic region according to Solon et al. [51].

To compare the effectiveness of LiDAR-UAV sensors in terms of topographic texture analyses, a dynamically developing small gully system constituting the southern branch of the Potok Stocki valley (Nałęczów Plateau, Lublin Upland, SE Poland) was selected (Figure 1). The sub-basin of the loess gully has an area of 1.3 km^2^ [50]. The investigated gully is located within a strongly dissected loess plateau, with a thickness of loess cover reaching 30 m [51].

Three LiDAR survey systems were used for the raids: YellowScan Mapper (YSM), CHC Alpha Air 450 (CHC) and DJI Zenmuse L1 (L1). The basic parameters of each system are summarised in Table 1.

Due to the significant forest cover of the gully system, flights were carried out before the beginning of vegetation (no foliage), on three dates: YSM—April 2021, CHC and L1—April 2022. It was assumed that changes in the geomorphology of the study area, if they even occurred, were of minor importance due to the management of the area (existing perennial forest cover). In each case, the altitude of the airstrike was set at 75–80 m above ground level (AGL). In addition, a fixed distance of UAV movement relative to the topographic surface was set in the mission planning software (following the model of the terrain). The total area covered by a single flight was approximately 1 km^2^. The Yellow Scan Mapper acquired the densest point cloud (Table 2).

**Figure 2:**
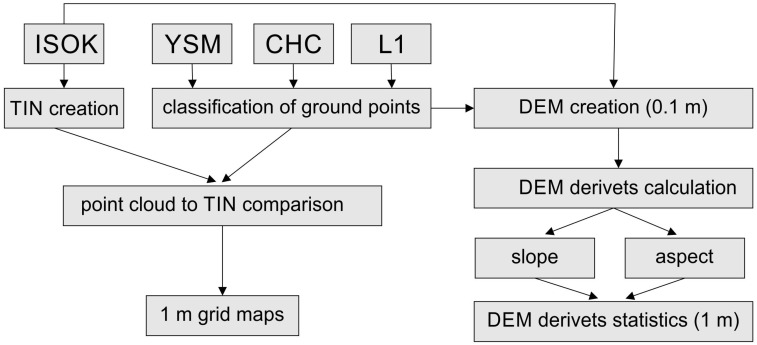
A flow chart of data processing and an indicators parameter calculation.

To compare the quality and relevance of the acquired data, a multi-stage framework was used to select ground points and develop high-resolution digital terrain models (DTMs) on their base; these were compared to the reference model and with each other. The following stages of data processing and benchmarking are shown in Figure 2.

The acquired raw data were classified in LP360 7.0 software (©GeoCue 520 6th Street, Madison, AL, USA) to extract the ground points. The acquired data has been pre-processed in the dedicated software of the respective manufacturer, based on the manufacturer’s recommended procedure. The data were imported into a common spatial reference system (EPSG:2178, PL-KRON86-NH). The area of interest (AoI) was then determined by taking the largest common area for all three raids, so that a direct statistical comparison of the data with each other is possible (Figure 1C).

Simultaneously, reference datasets were prepared. It was assumed that the reference point for all three LiDAR-UAV models would be airborne laser scanning (ALS) data acquired from the resources of the Central Office of Geodesy and Cartography (GUGiK), which were prepared within National Guard Information System project (ISOK). The data were acquired as point clouds classified according to the American Society for Photogrammetry and Remote Sensing (ASPRS) standard. They are made available to the public and free of charge via the online Geoportal platform (geoportal.gov.pl (accessed on 4 November 2022)). The ALS point cloud density for the analysed area ranges from 4 pts/m^2^ to 12 pts/m^2^. Based on the reference point cloud, a TIN model was created using the Delaunay triangulation method (2.5 D, best fitting plane), yielding a total of 1,399,244 triangles; interpolation was limited to 3m (value determined empirically). The analysis of individual LiDAR-UAV point clouds was carried out against a reference TIN model, prepared from ALS data, based on a 1m grid. On this basis, the number of points collected with a LiDAR-UAV lying on the ground in a given mesh was calculated. The difference in height recorded by the LiDAR-UAV relative to the reference TIN model was also determined statistically for each mesh − mean difference, median difference, standard deviation and variance.

In order to investigate the influence of the different sensors on the derived digital terrain models for the data from each sensor, digital terrain models with a resolution of 10 cm/pix were produced in LP360 software. Additionally, for each derived model, slope exposure and slope were calculated in QGIS 3.16 software. Using a 1 m resolution grid, the results were then compared using the root mean square deviation (RMSE) and relative root mean squared error (RRMSE) statistics.

**Figure 3:**
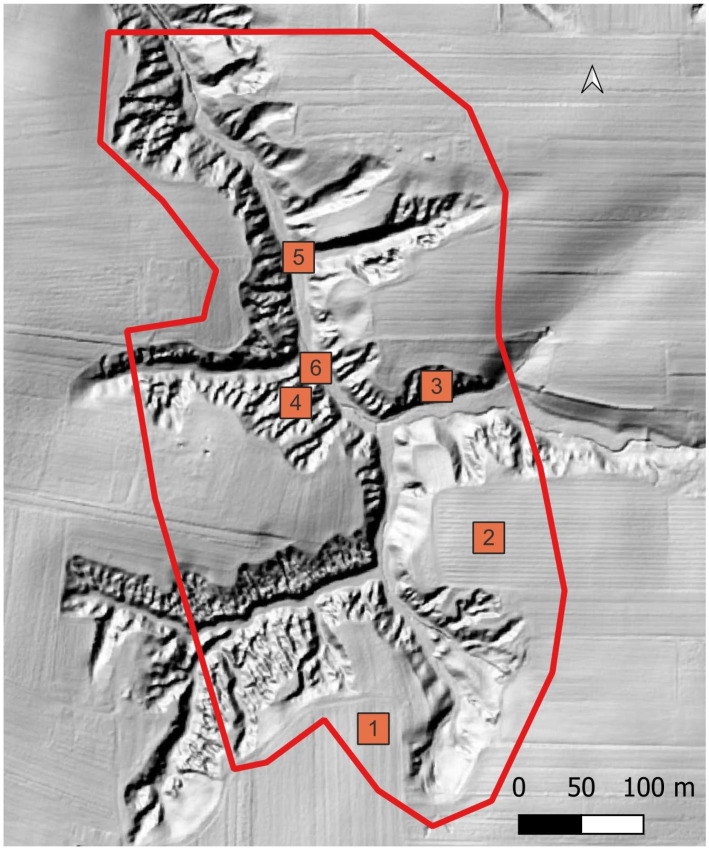
Location of test fields (525 m^2^). 1, 2—arable fields, 3, 4—locations on the slopes of the gully, 5, 6—locations in the bottom of the gully. Area of interest is marked with a red line.

In addition, test fields of ca. 500 m^2^ with a relatively uniform ground surface structure—cultivated field, ravine slope, ravine bottom—were delineated within the primary area of interest (Figure 3). The data from the individual sensors were compared in relation to the forms of use and statistically collated.

The acquired data were additionally validated by direct measurement on the ground, carried out using the GNSS-RTK method, at 24 points randomly distributed in the study area.

## 3. Results

### Comparison of Point Clouds to the Reference Model

Differences were found for all sensors while comparing the data acquired directly to the reference model created from the ALS data. A statistical summary is presented in Table 3.

**Figure 4:**
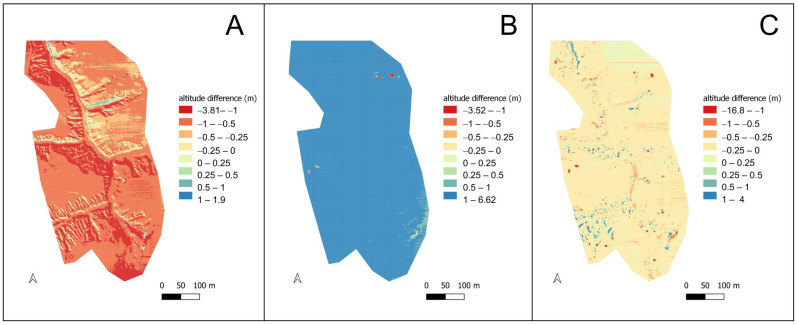
Differences in recorded altitude between LiDAR-UAV and the reference model. (**A**) YSM, (**B**) CHC, (**C**) L1.

For the entire study area, large differences from the reference model were recorded, with registered altitudes being higher over almost the entire study area (Figure 4A). It is noteworthy that the differences correspond very clearly to the relief and their visualisation resembles the DTM. In the area of the forested gully the differences reached over 1 m, within the cultivated fields they were in the range of 0.5–1 m.

Significant differences were also found for the CHC Alpha Air. In this case, significantly lower ground altitudes were recorded than on the reference model. The difference was systematic; for the whole area it was very significant, showing a slight relationship with the relief. The high positive values are spotty, associated with the development area in the north-eastern part of the study area (Figure 4B). 

For the DJI Zenmuse L1, the average difference between the recorded point and the height value read from the model was 13 cm, with a median of 12 cm. The high values of maximum and minimum difference are noteworthy. High positive differences (higher values recorded for LiDAR-UAV), reaching more than 20 metres, are found throughout the area and are mostly point and diffuse in nature (Figure 4C).

The pronounced negative differences (lower values recorded for LiDAR-UAV) are area-specific. Altitudes more than 2 m lower than the reference model were recorded at two locations. In the northern part of the area this is the bottom of the gully, in the southern end it is the edge of the gully immediately adjacent to the boundary of the arable field (Figure 4C).

A summary of the specific errors between the values determined from the LiDAR-UAV data and the reference model is summarised in Table 4. For the L1 scanner, the average error in height determination was 0.31 m, while the largest was for the CHC, reaching over 3 metres. For gradients, the highest error was estimated for the YSM, reaching over 40 degrees; for the other two sensors it was slightly in the region of 10 degrees. In the case of aspects, a significant error was also found for the YSM, at over 70 degrees, for the L1 and CHC it was also high, at around 50 degrees.

**Figure 5:**
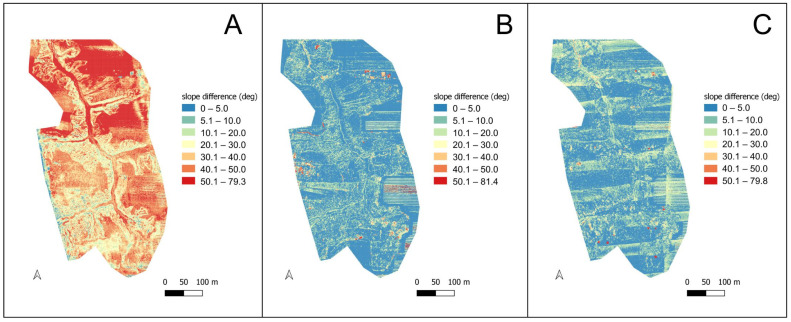
Difference in recorded slopes between LiDAR-UAV and the reference model. (**A**) YSM, (**B**) CHC, (**C**) L1.

Significant differences were recorded for the numerical terrain model derivatives, namely slopes and aspects. In the case of the YSM scanner, differences in recorded slopes were high throughout the study area, locally reaching nearly 80° (Figure 5A). Characteristically, smaller differences were found in areas with more varied relief, relative to relatively uniformly relief areas. The CHC sensor data also varied over a wide range, locally exceeding 80° but, nevertheless, the mean difference was at a much lower level and high discrepancies were found within the cultivated fields, clearly relating to the direction of cultivation, but also in the partially developed area in the north-eastern part of the site and in parts of the ravine system (Figure 5B). In the case of L1, significant differences were spotty, mainly related to anthropogenic objects (buildings), and the direction of cultivation was also reflected, but the variation was at a relatively low level (Figure 5C).

**Figure 6:**
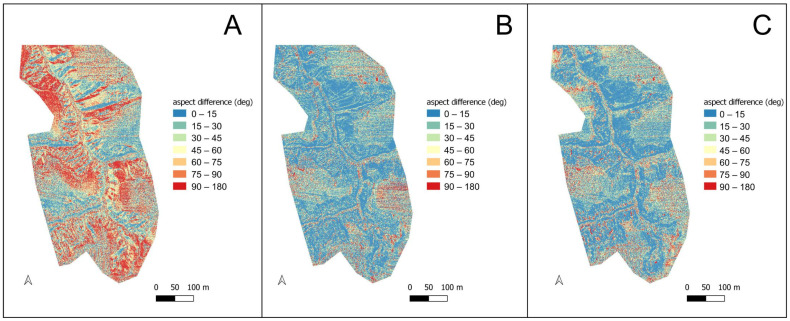
Difference in recorded aspects between LiDAR-UAV and the reference model. (**A**) YSM, (**B**) CHC, (**C**) L1.

In terms of the defining aspect, the differences in all cases reached 180°. In the case of YSM, they were high throughout the area (Figure 6A), both in the highly sculptured area and in locations with less variation in relief. Much lower divergences were found for CHC (Figure 6B), with the most significant differences occurring in the cultivated field and following the direction of cultivation. L1 also showed a point-to-point variation of 180°, mainly in locations associated with human activities; for this sensor, the divergence of the specific exposure from the reference model was the lowest (Figure 6C).

Within the designated additional test plots (cultivated field, gully slope, gully bottom), differences in individual separations were determined analogously to the analysis of the entire site. The differences of the selected parameters developed variously. In the case of absolute height, very high relative model convergence was recorded for the L1 cloud in each land use version. For the CHC sensor, significantly higher differences were found at the bottom of the gully; in the cultivated field—although large—they were lower. An inverse relationship was found for YSM—in the arable field the differences were half as high as at the bottom of the gully (Table 5).

## 4. Discussion

This study analyses the errors that can arise during the acquisition and processing of LiDAR data using a UAV platform. The large number of works using LiDAR-UAV data in recent years allows us to conclude that this type of data is widespread and used in many ways. However, despite the widespread use of this technology, a critical approach to both data acquisition and processing methods is required.

The significant positive differences in the case of the L1 scanner are mainly due to errors in the classification of the point cloud, individual trees were classified in class 2 points lying on the ground. A similar situation occurred for buildings in the eastern part of the study area, where the roofs of buildings were also classified as ground. An important factor may be the configuration of the point cloud classification software which, in the case of the site analysed here, must take into account the varying relief and use of the scanned area. Differences can occur in arable fields where, despite the relatively smooth slopes of the flat surface, the whole area is subject to seasonal variations in elevation due to tillage operations. On the other hand, the edges of the gully, the steep slopes and the flat bottom of the gully are subject to erosion and deposition processes, and seasonal differences here can reach several to tens of centimetres. Areas with significant coverage of tall vegetation, parts of buildings, etc., are also difficult to classify. The issue of classification of points lying on the ground in forested areas has been the subject of many studies [52,53], but it is not the subject of this paper.

It is worth highlighting that the large discrepancies in values occur basically only in the forested area, with a well-developed vegetation cover. The exception is the mentioned building part. The density of the base cloud is an important factor; for the reference data the average density is low, for areas with developed vegetation the number of points is further limited in the classification process.

The height interpolation used, carried out on a rare cloud, may not take into account small landforms and thus apparent differences between the LiDAR-UAV data and the reference model are generated.

In the case of large negative differences, it should be taken into account that the ALS data acquisition took place in 2011, a difference of more than a decade for an active gully form is reflected in the recorded height difference (mainly in the gully axes). This could be an important contribution to the use of LiDAR-UAV data in monitoring geomorphological change on a local scale, using data acquired over a large time interval.

In the case of the YSM cloud, no relevant differences were found that could be related to classification errors (misclassification of points from other classes to points lying on the ground). However, the spatial distribution of the differences is interesting; there is a clear relationship with land exposure, with the highest values recorded on slopes with northern and north-eastern orientation.

**Figure 7:**
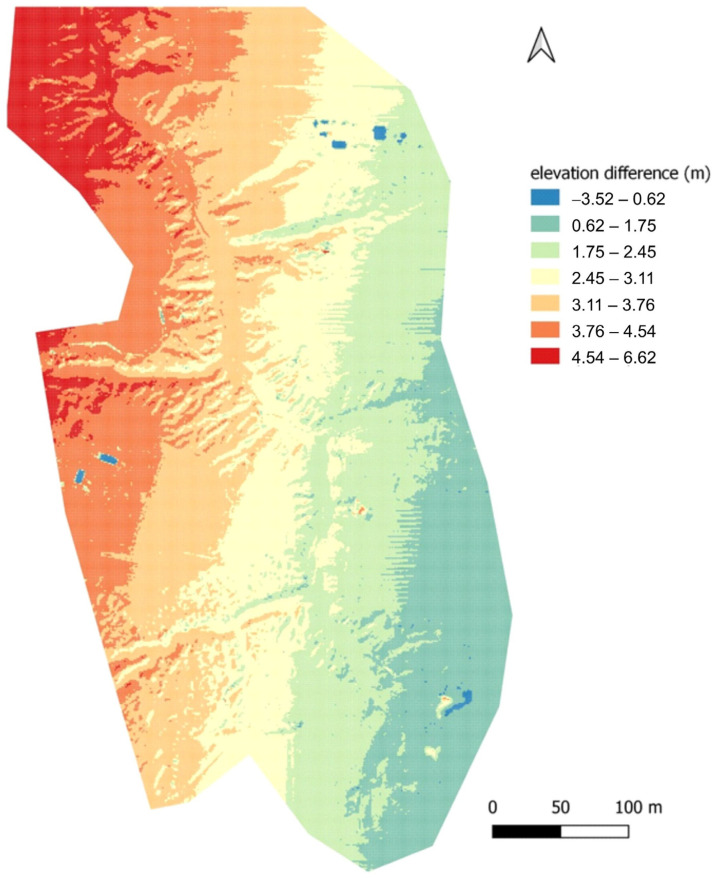
Differences in recorded altitude between LiDAR-UAV–CHC. Custom scale was used.

In the case of CHC, the differences from the reference model did not show a relationship with location relative to relief. Errors related to the misclassification of the point cloud were spotty, with the classification algorithm misassigning a class for fragments with buildings. However, a large error of a systematic nature appears for this dataset, evident when using a custom scale for data visualisation (Figure 7).

The clear, unambiguous meridional pattern of values may indicate erroneous IMU readings during data acquisition; the raid was performed in the north–south axis, hence the error may be related to an incorrect reading of roll axis excursions. Similar conclusions were reached by Huising and Pereira [54] analysing data from different sensors. 

Differences in recorded terrain altitude, relative to a specific reference model, can be a subject of discussion in itself.The values of possible data recording accuracy, indicated by the manufacturers of individual systems, may suggest a high reliability of the data; however, the verification carried out shows significant discrepancies. Kamp et al. [55] presented a summary of possible errors in LiDAR-based ground surface measurements. They also made a direct comparison between LiDAR-UAV data and ALS data; however, an ALS and LiDAR-UAV matching was performed on the basis of selected flat surfaces and the ALS cloud was used for additional height adjustment. Differences between models developed from LiDAR-UAV and other sources were signalled by de Magalhães and Moura [56], who conducted their study in an urbanised area. The registration of incorrect values may be related to errors in the UAV’s IMU or the incorrect determination of the UAV’s flight trajectory [57]. Differences between the recorded value and the actual value may increase with flight altitude [58], which is confirmed by the results obtained; a flight altitude of 75 m is relatively high for LiDAR-UAV acquisition.

In the context of the results obtained, it is essential to carry out checks by direct GNSS measurement on the ground and to make any elevation corrections each time. Room et al. [59] signal that it is necessary to use GCPs, also with LiDAR-UAV, and that the number of points used is proportional to the quality of the model obtained. When using control points, Bakuła et al. [57] achieved high convergence between LiDAR-UAV data and the reference model, using the same data source for comparison (ALS). In the case of the present study, GCPs were used as checkpoints, not control points, to verify absolute heights. The use of GCPs, however, is not required by the providers of the particular systems.

When using LiDAR-UAV data for local studies, the recorded differences may not be of great importance, as the differences in relative heights remain at a similar, acceptable level. LiDAR-UAVs have been used in recent years for crop height estimation [14], where estimating the height difference over a specific time interval is crucial; in such studies, absolute height is less important.

Bartholomeus et al. [60] undertook a similar study, comparing data acquired from three different systems. The study was conducted in a forested area, but with a low crown density. Despite this, they recorded height differences of up to several tens of centimetres between the different systems. One has to agree and support their conclusion that in areas of greater complexity the differences can be much higher, which was confirmed in the present study. However, similar comparative studies are still lacking in the literature; it is to be expected that technological developments will force analogous research work in the near future.

Comparative studies conducted in a terrain with quite varied relief, which the Nałęczów Plateau undoubtedly is, are a novelty and need confirmation in other research areas. The combination of the influence of the relief with the presence of dense thickets and trees makes the data from this area require much more attention and the application of the latest classification algorithms than in the case of flat areas covered only with low vegetation. Therefore, further research is needed to improve mission design in such terrain, as well as classification algorithms.

## 5. Conclusions

- A comparison of elevation data acquisition LiDAR-UAV systems shows large discrepancies between the various systems, exceeding the figures quoted by manufacturers. The maximum differences for the YSM scanner reached 3.80 m, for CHC they were 6.62 m, for L1 they reached 16.8 m. The high differences were mainly due to misclassification of point clouds;
- For proper data acquisition, it is necessary to use reference data, acquired by traditional techniques (ground-based GNSS surveys), to ensure the quality of the final product;
- In the heavily textured and forested area, the differences in registered altitude for the CHC scanner were much higher than for the less diverse area (3.3 vs. 2.0 m). For YSM, the relationship was opposite, but the differences were at a lower level (about 0.2 m). For L1, the differences between the differentiated survey fields were insignificant;
- The use of data with different resolutions results in errors due to the relief of the terrain and a randomness in the location of height points on the LiDAR-UAV data;
- Due to technical considerations, relatively small areas can be subjected to LiDAR-UAV analyses compared to ALS data;
- Future research should expand the range of sensors used; further comparative studies of different market solutions are advisable.

## Figures and Tables

**Table 1:** Technical specifications of the LiDAR-UAV systems; data from manufacturers’ technical specifications.

Parameter/Sensor	YSM	CHC	L1
Scanner	Livox Horizon	Livox avia	Livox avia
Wavelength	905 nm	905 nm	905 nm
Precision (1)	2 cm	1.5 cm	1.5 cm
Accuracy (2)	3 cm @ 50 m AGL	2 cm @ 20 m AGL 10 cm @ 100 m AGL	Hz: 10 cm @ 50 m AGL V: 5 cm @ 50 m AGL
Point Range	Up to 240 k	570 pts/sqm @ 50 m AGL 280 pts/sqm @ 100 m AGL *	Single return: 240 k pts/s Multiple return: 480,000 pts/s
Shots per second	240 k	240–720 k	240–720 k
Echoes per shot	Up to 2	Up to 3	3
Scanner field of view FOV	81.7°	70.4°	95°
Inertial solution update rate	200 Hz (Applanix APX-15 UAV)	500 Hz	200 Hz
Weight	1.5 kg	0.95 kg	0.9 kg
Size	L 14.3 × W 9.5 × H 15.4 cm	12.8 × 12.8 × 6.75 cm	15.2 × 11.0 × 16.9 cm
Operating temperature	−20 to +40 °C	−20 °C to +50 °C	−20 °C to +50 °C
IP	-	64	54
Supported Aircraft	Matrice 300 RTK	Matrice 300 RTK	Matrice 300 RTK

(1) Precision, also called reproducibility or repeatability, accounts for the variation in successive measurements taken on the same target. (2) Accuracy is the degree of conformity of a measured position to its actual (true) value. * Point density on UAV setup 5 m/s (18 km/h) speed.

**Table 2:** Characteristics of the point clouds analysed. Values of the basic statistics of the LiDAR-UAV point clouds refer to 1m of mesh, created from ALS reference data.

	YSM	CHC	L1	ALS
Total number	86,451,462	34,751,385	4,558,276	700,706
Mean	91.14325	71.34123	22.97289	-
Max	100	100	98	-
Min	0	0	0	-
Median	48.57162	76	21	-

**Table 3:** Overview of the differences found between the point cloud acquired with the individual sensors and the reference model. Values in m.

	YSM	CHC	L1
Mean	−0.77	2.67	−0.13
Max	1.90	6.62	3.99
Min	−3.80	−3.52	−16.8
Median	−0.72	2.63	−0.12

**Table 4:** RMSE for individual sensors, determined in relation to the reference model.

	L1	YSM	CHC
Elevation (m)	0.31	0.87	3.11
Slope (degree)	9.3	42.6	10.0
Aspect (degree)	51.7	71.8	48.7

**Table 5:** Differences in selected parameters within the selected test fields.

Parameter	Sensor	Arable Fields		Slopes of the Gully		Bottom of the Gully	
		Mean	Median	Mean	Median	Mean	Median
Elevation (m)	CHC	1.9	2.0	2.6	2.7	3.3	3.3
	L1	0.1	0.1	0.2	0.1	0.1	0.1
	YSM	0.9	0.8	0.7	0.7	0.6	0.6
Slope (deg)	CHC	10.3	4.2	1.7	0.8	3.3	1.9
	L1	10.3	4.2	1.7	0.8	3.3	1.9
	YSM	34.4	35.2	27.5	29.2	40.1	41.9
Aspect (deg)	CHC	21.5	19.1	4.1	1.2	18.1	6.1
	L1	21.5	19.1	4.1	1.2	18.1	6.1
	YSM	26.8	29.6	16.8	12.0	23.5	18.8

## Data Availability

The data presented in this study are available on request from the corresponding author. The data are not publicly available due to data volume size and technical repository limitations.
